# Supplementary material for: Somatic mutation rates scale with lifespan across mammals
Source: Nature. 2022 Apr 13;604(7906):517–24. doi: 10.1038/s41586-022-04618-z (PMC9021023; doi:10.1038/s41586-022-04618-z)
Supplement: Supplementary file 1 — This file contains Supplementary Note 1 (Theoretical considerations on ageing), Supplementary Note 2 (Cancer risk modulation in the Armitage–Doll model), Supplementary References, Supplementary Figures 1–2, and legends for Supplementary Tables 1–7. [file 41586_2022_4618_MOESM1_ESM.pdf]

nature portfolio

https://doi.org/10.1038/s41586-022-04618-z

Supplementary information

Supplementary Information for

Somatic mutation rates scale with lifespan across mammals

Alex Cagan, Adrian Baez-Ortega, Natalia Brzozowska, Federico Abascal, Tim H. H. Coorens, Mathijs A. Sanders, Andrew R. J. Lawson, Luke M. R. Harvey, Shriram Bhosle, David Jones, Raul E. Alcantara, Timothy M. Butler, Yvette Hooks, Kirsty Roberts, Elizabeth Anderson, Sharna Lunn, Edmund Flach, Simon Spiro, Inez Januszczak, Ethan Wrigglesworth, Hannah Jenkins, Tilly Dallas, Nic Masters, Matthew W. Perkins, Robert Deaville, Megan Druce, Ruzhica Bogeska, Michael D. Milsom, Björn Neumann, Frank Gorman, Fernando Constantino-Casas, Laura Peachey, Diana Bochynska, Ewan St. John Smith, Moritz Gerstung, Peter J. Campbell, Elizabeth P. Murchison, Michael R. Stratton, Iñigo Martincorena

Table of contents

Supplementary Note 1. Theoretical considerations on ageing …..…………………………… 2
Supplementary Note 2. Cancer risk modulation in the Armitage–Doll model ……………… 7
Supplementary Figures …...…………..…………..…………..…………..………………… 12
Supplementary Table legends ……………..…………………..…………..………………… 14

Supplementary Note 1. Theoretical considerations on ageing

Here we provide some theoretical considerations on the evolution of ageing and the multifactorial nature of ageing that can help interpret and contextualise our findings.

Evolutionary and mechanistic theories of ageing

Multiple forms of molecular damage accumulate in cells over time and have been proposed to contribute to ageing. They include somatic mutations, telomere attrition, epigenetic drift and loss of proteostasis, among others[1]. The rates of accumulation of molecular damage cannot be uncontrolled, or else they could compromise survival and reproduction. This has led to the evolution of multiple repair mechanisms against different forms of damage. However, as we explain below, two main evolutionary factors limit the extent to which repair mechanisms can evolve to reduce the rates of molecular and tissue damage, and thereby to increase lifespan: weak selection and evolutionary trade-offs[2–6].

The first factor limiting the evolution of repair mechanisms is weak selection for longer lifespans than typical in the wild. Building on ideas from Fisher and Haldane, in 1952 Medawar explained how selection is unable to remove germline mutations from a population if their deleterious effects manifest after the vast majority of individuals have already died (a concept known as the "selection shadow"). Medawar's "mutation accumulation" theory of ageing (referring to germline mutations) thus proposed that ageing could result from the accumulation in a species of deleterious germline mutations whose effects manifest late in life[2]. External causes of death such as predation, infection or starvation determine the average lifespan of individuals of any species in the wild. Selection will favour adaptations that reduce premature death from intrinsic causes in the wild (*e.g.* cancer or ageing), such as reductions in the rates of molecular damage accumulation, but these adaptations can only reach fixation in a population (overcoming the genetic drift barrier[7]) if enough individuals benefit from them. As a result, selection for improved repair mechanisms is ineffective in delaying ageing too far beyond the typical lifespan of a species in the wild. This argument predicts that species with a lower extrinsic mortality (*i.e.* longer average lifespan in the wild) will evolve better molecular repair mechanisms and longer maximum lifespans. As a result of selection acting on the rates of repair, the rates of accumulation of molecular damage are expected to vary widely across species and result in an inverse relationship with lifespan (*i.e.* a similar burden of damage at the end of lifespan), as we report for somatic mutation rates in the present study.

A second evolutionary limit is that, in maximising reproductive success, evolution can favour adaptations that increase reproduction at the cost of a shorter lifespan (Williams's "antagonistic pleiotropy" theory of ageing[3]). An example of this argument is the "disposable soma" theory of ageing, which proposes that there is a trade-off between energy investment in repair and reproduction[8,9]. Together, genetic drift and evolutionary trade-offs predict that organisms in the wild will tend to die young from extrinsic causes, while organisms in protected environments (with limited extrinsic mortality) will live considerably longer but tend to succumb to ageing and age-related diseases, as a result of the accumulation of molecular and tissue damage to which the species has not evolved effective response strategies.

Multifactorial nature of ageing

The evolutionary limits to the evolution of repair mechanisms apply similarly to any form of molecular or tissue damage that accumulates with age and that could lead to premature death if its rate were uncontrolled. Indeed, similar anticorrelations with lifespan have been reported for other forms of damage, such as telomere shortening rates[10] and loss of protein stability[11]. As noted by Maynard Smith in 1962[5], a consequence of selection acting on the rates of somatic damage is that the phenotypic manifestations of different forms of damage can appear semi-synchronously late in life, even if they occur independently. This predicts that ageing is likely to be multifactorial, with the consequences of multiple forms of molecular and tissue damage expected to manifest late in life. Furthermore, different forms of molecular and tissue damage are likely to synergise, causing a faster functional decline than they would achieve independently.

These evolutionary considerations imply that ageing is likely multifactorial, in line with the current mechanistic understanding of ageing[5], with multiple forms of molecular damage being likely responsible for different ageing phenotypes. In this context, the question of whether somatic mutations *cause* ageing could be misleadingly simplistic. A more appropriate question may be to what extent, *if any*, and through which precise mechanisms somatic mutations contribute to different age-related diseases or phenotypes.

Deleterious mutations *versus* selfish mutant clones in ageing tissues

Discussions on the role of somatic mutations in ageing often assume that somatic mutations are deleterious to the carrying cell, contributing to ageing by causing loss of cellular fitness or cell death[12,13]. The earliest models of ageing by somatic mutation also assumed that the rate of somatic

mutation accelerates with age, as somatic mutations accumulate and erode DNA repair pathways[12]. Recent sequencing studies of human tissues have largely ruled out these models. First, data across tissues suggest that the increase of somatic mutations with age is approximately linear, meaning that the somatic mutation rate is roughly constant across life[14,15]. Second, mutation rates in most human tissues are on the order of 15–45 mutations per diploid genome per year, which appears much lower than would be required to cause biallelic inactivation of important genes in a relevant fraction of cells in elderly individuals[14–16]. Third, dN/dS analyses of selection acting on somatic mutations in cancer genomes and normal tissues have revealed that even mutations affecting protein-coding genes (including missense and nonsense mutations) appear to be tolerated by somatic cells, with dN/dS ratios ~1 implying that the vast majority of coding mutations do not cause somatic cell death or impaired proliferation[17,18]. Fourth, somatic mutation studies of individuals with hypermutator phenotypes (*e.g. POLE*/*POLD1* or *MUTYH* mutations) have revealed that somatic cells can tolerate large increases in somatic mutation rates[19,20]. Given these considerations, it appears increasingly unlikely that somatic mutations could contribute significantly to ageing by directly causing cell death or impaired proliferation.

An alternative model of how somatic mutations could contribute to ageing is through positive selection on certain mutations causing clonal expansions of phenotypically aberrant cells. This possibility was discussed by some early authors[5,21–23], but has acquired particular significance in the last few years, in light of the widespread evidence of colonisation of some somatic tissues by clones carrying positively-selected (driver) mutations[24–27]. This phenomenon has been observed in tissues such as blood[24,28], skin[25], oesophagus[26], endometrium[29], lung[30] and bladder[31], with a considerable fraction of all cells in these tissues carrying a positively-selected somatic mutation, often in known cancer genes. The clinical relevance of these clones for diseases other than cancer remains largely unstudied, but since somatic selection operates at the level of cells rather than tissues or organisms, it is likely that selfishly selected clones are more often deleterious than beneficial to the organism.

Positively-selected somatic mutations in stem cells would naturally favour proliferation over differentiation[32] and could lead to biased cell fates, resulting in cell type imbalances[33]. Selection for increased proliferation in tissues maintained by self-duplication of differentiated cells could favour de-differentiation, loss of functional specialisation and reduced production of key protein products. For example, selection of certain driver mutations in haematopoietic stem cells can lead to mutant clones colonising the haematopoietic system, a common phenomenon in old age known as “clonal haematopoiesis”. Some of these mutations alter differentiation trajectories and lead to

cell type imbalances[33]. Clonal haematopoiesis has also been associated with the risk of cancer and cardiovascular disease[24]. Positively selected somatic mutations in key metabolic pathways have been reported in the context of chronic liver disease, with mutations causing insulin resistance or altered lipid metabolism benefiting the mutant cells, probably at a cost to the organism[34]. Somatic driver mutations have also been reported to contribute to the rapid growth of some cavernomas in the brain, leading to seizures and strokes[35].

In summary, while somatic point mutations appear unlikely to significantly contribute to ageing through direct deleterious effects on the mutant cells, positive selection of selfish mutant clones offers a plausible mechanism by which somatic mutation could contribute to ageing. Such a mechanism might also operate through somatically heritable epigenetic changes.

The hypermutator paradox

Two recent studies have measured somatic mutation rates in individuals with familial cancer predisposition syndromes caused by germline mutations in the DNA polymerases Pol ε or Pol δ (*POLE*/*POLD1*) or in the base excision repair DNA glycosylase MUTYH[19,20]. These syndromes are associated with a marked increase in the risk of colorectal and endometrial cancer. Measurement of somatic mutation rates in normal colon and endometrium in these patients revealed point mutation rates several-fold higher than those of unaffected individuals. Intriguingly, these increases were not associated with overt evidence of accelerated ageing in these patients[19,20].

These results largely rule out a simple somatic mutation model of ageing, whereby the mutation burden is solely responsible for all the varied manifestations of ageing. However, these findings need to be interpreted with caution and do not rule out a possible contribution of somatic mutations to ageing. Although genome-wide somatic mutation rates were several-fold higher in the colonic and endometrial epithelium in these donors, the increase in mutation rates in protein-coding regions of the genome in less mitotic tissues appeared to be more modest (<2 fold). The cohorts of individuals with these cancer predisposition syndromes are also small and not sufficiently characterised to rule out modest increases of some age-related diseases. In addition, the theoretical considerations above may also help explain this paradox. First, as discussed above, ageing is likely to be multifactorial. Increasing the rate of a single form of damage, such as somatic mutations, is not expected to accelerate ageing linearly or impact all ageing phenotypes. An increase in mutation rates may have little or no impact on age-related phenotypes largely caused by other forms of damage, or on phenotypes that result from the interaction of somatic mutations and other forms of

damage. The multifactorial nature of ageing can also help explain why experimental shortening of telomeres or increases in mitochondrial mutation rates do not result in a linear acceleration of all or most ageing phenotypes[36–38]. Second, the possibility that somatic mutations may contribute to organismal ageing via clonal expansions can introduce considerable non-linearity in the consequences of increasing somatic mutation rates. For example, doubling the somatic mutation rate in haematopoietic stem cells may double the rate of occurrence of driver mutations, but their phenotypic manifestations will be delayed by the need for these clones to grow to a clinically relevant size, a process that can take decades in humans[39].

Overall, the observation that individuals with germline *POLE*, *POLD1* or *MUTYH* mutations have increased somatic mutation rates without an overt accelerated ageing syndrome presents a paradox that deserves careful investigation, but it does not rule out a possible role for somatic mutations in ageing. Sequencing studies on individuals carrying DNA repair defects causing progeroid syndromes could help shed additional light on this question. Further, detailed studies of the extent and impact of somatic mutations in individual age-related diseases and phenotypes would be required to obtain a more mechanistic and definitive understanding of the possible links between somatic mutations and ageing.

Altogether, the observation of a strong scaling of somatic mutation rates and lifespan across 16 mammalian species, despite variable contributions of different mutational processes across species, is consistent with somatic mutation rates being evolutionary constrained, and with somatic mutations contributing to some extent to organismal ageing. This interpretation is further supported by studies reporting improved DNA repair and genome maintenance in longer-lived species[40–43]. However, alternative interpretations for the inverse relationship between somatic mutation rates and lifespan may be possible.

Supplementary Note 2. Cancer risk modulation in the Armitage–Doll model

In the early 1950s, analysing human cancer mortality data across cancer types, several authors observed that cancer incidence increases approximately geometrically with age to the power of six. Armitage and Doll[44], building on the ideas of Nordling[45], proposed that such observation was consistent with a model in which a normal cell needs to acquire 7 driver mutations to transform into a cancer cell. Under this simple model, assuming that somatic mutations occur approximately linearly with age (as it has now been confirmed across multiple tissues) and that the occurrence of each mutation is a relatively rare event, the incidence of cancer at age t is proportional to the probability of each mutation per unit of time and the sixth power of the age:

$$\text{cancer rate} = k\, p_1\, p_2\, p_3\, p_4\, p_5\, p_6\, p_7\, t^6,$$

k being a constant term. This model can be expressed as a function of the somatic mutation rate per base pair, assuming that the probability of each driver mutation is proportional to the somatic mutation rate per base pair, u, and the target size and mutability of each driver gene, a:

$$\text{cancer rate} = k\, a_1 u\, a_2 u\, a_3 u\, a_4 u\, a_5 u\, a_6 u\, a_7 u\, t^6.$$

The model can be generalised by assuming a requirement of n driver mutations, with k' being a different constant term including the product of the a terms:

$$\text{cancer rate} = k'\, u^n\, t^{n-1}.$$

The Armitage and Doll model, often referred to as the multistage model of carcinogenesis, is simplistic in many ways, but remains an influential model and a useful and simple tool to discuss the ability to modify cancer risk by changes in either the somatic mutation rate or the number of driver mutations required to transform a normal cell into a cancer cell.

Using this model, we can compare the effect on the cancer rate of reducing the mutation rate per year by a factor d. Under the model above, the fold change in cancer rate is given by

$$\frac{k' \left(\frac{u}{d}\right)^n t^{n-1}}{k'\, u^n t^{n-1}} = \frac{\frac{u^n}{d^n}}{u^n} = \frac{1}{d^n}.$$

Therefore, reducing the mutation rate per year by a factor of d causes the cancer rate to decrease by a factor of d^n. For instance, with $n = 6$ drivers for colorectal cancer[44], we obtain that halving the mutation rate ($d = 2$) reduces the cancer risk by a factor of $2^6 = 64$, independently of age.

In contrast, increasing the required number of driver mutations from n to $n + m$ leads to the following fold change in the cancer rate:

$$\frac{k'\ a_{n+1} \ldots a_{n+m}\ u^{n+m} t^{n+m-1}}{k'\ u^n t^{n-1}} = a_{n+1} \ldots a_{n+m}\ (ut)^m.$$

For example, a species could add an additional barrier to tumour formation by duplicating a tumour suppressor gene whose mutation is essential for cancer development or by strengthening existing tumour suppression mechanisms. Using the mean ELB across species, normalised by genome size, leads to an approximate mutation burden per bp at the end of lifespan of $ut \sim 10^{-6}$ mutations per diploid bp. If we assume that a new tumour suppressor gene has $a = 100$ sites where a protein-truncating mutation can occur, the addition of this single additional cancer gene can in theory reduce cancer risk by a factor of $\sim 10^4$. This reduction could be larger if the new gene has a smaller target size (*e.g.* oncogenes) or requires two hits for inactivation, as is common in many tumour suppressor genes. On the other hand, tumours can evolve through mutation of different pathways, and duplication of an important tumour suppressor gene is likely to suppress just some of the routes by which normal cells can transform into cancers, likely achieving less dramatic reductions in cancer risk.

Although Armitage and Doll's multistage model is simplistic, it exemplifies how considerably large changes in cancer risk could be achieved in evolution by changes in the somatic mutation rate and/or the number of driver mutations required to transform a normal cell into a cancer cell. This general conclusion extends to more sophisticated models where the cancer risk remains a function of the probability of driver mutation per unit time raised to the number of required drivers, such as the model by Calabrese and Shibata[46,47]. These models help to explain the observation that cancer mortality risk is largely independent of both body mass and adult life expectancy across mammals, as recently demonstrated by a study of 191 mammalian species[48].

References

1. López-Otín, C., Blasco, M. A., Partridge, L., Serrano, M. & Kroemer, G. The hallmarks of aging. *Cell* **153**, 1194–1217 (2013).
2. Medawar, P. B. *An Unsolved Problem of Biology: An Inaugural Lecture Delivered at University College, London, 6 December, 1951.* (H.K. Lewis and Company, 1952).
3. Williams, G. C. Pleiotropy, Natural Selection, and the Evolution of Senescence. *Evolution* **11**, 398–411 (1957).
4. Charlesworth, B. Fisher, Medawar, Hamilton and the evolution of aging. *Genetics* **156**, 927–931 (2000).
5. Smith, J. M. Review Lectures on Senescence. I. The Causes of Ageing. *Proceedings of the Royal Society of London. Series B, Biological Sciences* **157**, 115–127 (1962).
6. Partridge, L. & Barton, N. H. Optimality, mutation and the evolution of ageing. *Nature* **362**, 305–311 (1993).
7. Lynch, M. The lower bound to the evolution of mutation rates. *Genome Biol. Evol.* **3**, 1107–1118 (2011).
8. Kirkwood, T. B. Evolution of ageing. *Nature* **270**, 301–304 (1977).
9. Kirkwood, T. B. & Holliday, R. The evolution of ageing and longevity. *Proc. R. Soc. Lond. B Biol. Sci.* **205**, 531–546 (1979).
10. Whittemore, K., Vera, E., Martínez-Nevado, E., Sanpera, C. & Blasco, M. A. Telomere shortening rate predicts species life span. *Proc. Natl. Acad. Sci. U. S. A.* **116**, 15122–15127 (2019).
11. Swovick, K. *et al.* Interspecies Differences in Proteome Turnover Kinetics Are Correlated With Life Spans and Energetic Demands. *Mol. Cell. Proteomics* **20**, 100041 (2021).
12. Szilard, L. On the nature of the aging process. *Proc. Natl. Acad. Sci. U. S. A.* **45**, 30–45 (1959).
13. Morley, A. A. The somatic mutation theory of ageing. *Mutat. Res.* **338**, 19–23 (1995).
14. Blokzijl, F. *et al.* Tissue-specific mutation accumulation in human adult stem cells during life. *Nature* **538**, 260–264 (2016).
15. Abascal, F. *et al.* Somatic mutation landscapes at single-molecule resolution. *Nature* (2021) doi:10.1038/s41586-021-03477-4.
16. Moore, L. *et al.* The mutational landscape of human somatic and germline cells. *Nature* **597**, 381–386 (2021).
17. Martincorena, I. *et al.* Universal Patterns of Selection in Cancer and Somatic Tissues. *Cell* **171**, 1029–1041.e21 (2017).
18. Lee-Six, H. *et al.* Population dynamics of normal human blood inferred from somatic mutations. *Nature* **561**, 473–478 (2018).
19. Robinson, P. S. *et al.* Increased somatic mutation burdens in normal human cells due to defective DNA polymerases. *Nat. Genet.* **53**, 1434–1442 (2021).

20. Robinson, P. S. *et al.* Inherited MUTYH mutations cause elevated somatic mutation rates and distinctive mutational signatures in normal human cells. *bioRxiv* 2021.10.20.465093 (2021) doi:10.1101/2021.10.20.465093.
21. Burnet, M. Intrinsic mutagenesis. (1974) doi:10.1007/978-94-011-6606-5.
22. Burnet, M. Somatic mutation and chronic disease. *Br. Med. J.* **1**, 338–342 (1965).
23. Burch, P. R. J. Possible Aetiological Significance of the Age-Pattern of Certain Diseases. *Postgrad. Med. J.* **40**, 151–153 (1964).
24. Jaiswal, S. *et al.* Age-related clonal hematopoiesis associated with adverse outcomes. *N. Engl. J. Med.* **371**, 2488–2498 (2014).
25. Martincorena, I. *et al.* Tumor evolution. High burden and pervasive positive selection of somatic mutations in normal human skin. *Science* **348**, 880–886 (2015).
26. Martincorena, I. *et al.* Somatic mutant clones colonize the human esophagus with age. *Science* **362**, 911–917 (2018).
27. Mustjoki, S. & Young, N. S. Somatic Mutations in 'Benign' Disease. *N. Engl. J. Med.* **384**, 2039–2052 (2021).
28. Genovese, G. *et al.* Clonal hematopoiesis and blood-cancer risk inferred from blood DNA sequence. *N. Engl. J. Med.* **371**, 2477–2487 (2014).
29. Moore, L. *et al.* The mutational landscape of normal human endometrial epithelium. *Nature* **580**, 640–646 (2020).
30. Yoshida, K. *et al.* Tobacco smoking and somatic mutations in human bronchial epithelium. *Nature* **578**, 266–272 (2020).
31. Lawson, A. R. J. *et al.* Extensive heterogeneity in somatic mutation and selection in the human bladder. *Science* **370**, 75–82 (2020).
32. Bodmer, W. F. & Crouch, D. J. M. Somatic selection of poorly differentiating variant stem cell clones could be a key to human ageing. *J. Theor. Biol.* **489**, 110153 (2020).
33. Izzo, F. *et al.* DNA methylation disruption reshapes the hematopoietic differentiation landscape. *Nat. Genet.* **52**, 378–387 (2020).
34. Ng, S. W. K. *et al.* Convergent somatic mutations in metabolism genes in chronic liver disease. *Nature* **598**, 473–478 (2021).
35. Ren, A. A. *et al.* PIK3CA and CCM mutations fuel cavernomas through a cancer-like mechanism. *Nature* **594**, 271–276 (2021).
36. Blasco, M. A. The telomerase knockout mouse. in *Advances in Cell Aging and Gerontology* vol. 8 151–165 (Elsevier, 2001).
37. Vermulst, M. *et al.* Mitochondrial point mutations do not limit the natural lifespan of mice. *Nat. Genet.* **39**, 540–543 (2007).
38. Khrapko, K. & Vijg, J. Mitochondrial DNA mutations and aging: a case closed? *Nature genetics* vol. 39 445–446 (2007).

39. Fabre, M. A. *et al.* The longitudinal dynamics and natural history of clonal haematopoiesis. *bioRxiv* 2021.08.12.455048 (2021) doi:10.1101/2021.08.12.455048.
40. Hall, K. Y., Hart, R. W., Benirschke, A. K. & Walford, R. L. Correlation between ultraviolet-induced DNA repair in primate lymphocytes and fibroblasts and species maximum achievable life span. *Mech. Ageing Dev.* **24**, 163–173 (1984).
41. MacRae, S. L. *et al.* DNA repair in species with extreme lifespan differences. *Aging* **7**, 1171–1184 (2015).
42. Tian, X. *et al.* SIRT6 Is Responsible for More Efficient DNA Double-Strand Break Repair in Long-Lived Species. *Cell* **177**, 622–638.e22 (2019).
43. Zhang, L. *et al.* Maintenance of genome sequence integrity in long- and short-lived rodent species. *Sci Adv* **7**, eabj3284 (2021).
44. Armitage, P. & Doll, R. The age distribution of cancer and a multi-stage theory of carcinogenesis. *Br. J. Cancer* **8**, 1–12 (1954).
45. Nordling, C. O. A new theory on cancer-inducing mechanism. *Br. J. Cancer* **7**, 68–72 (1953).
46. Calabrese, P. & Shibata, D. A simple algebraic cancer equation: calculating how cancers may arise with normal mutation rates. *BMC Cancer* **10**, 3 (2010).
47. Caulin, A. F., Graham, T. A., Wang, L.-S. & Maley, C. C. Solutions to Peto's paradox revealed by mathematical modelling and cross-species cancer gene analysis. *Philos. Trans. R. Soc. Lond. B Biol. Sci.* **370**, (2015).
48. Vincze, O. *et al.* Cancer risk across mammals. *Nature* **601**, 263–267 (2022).

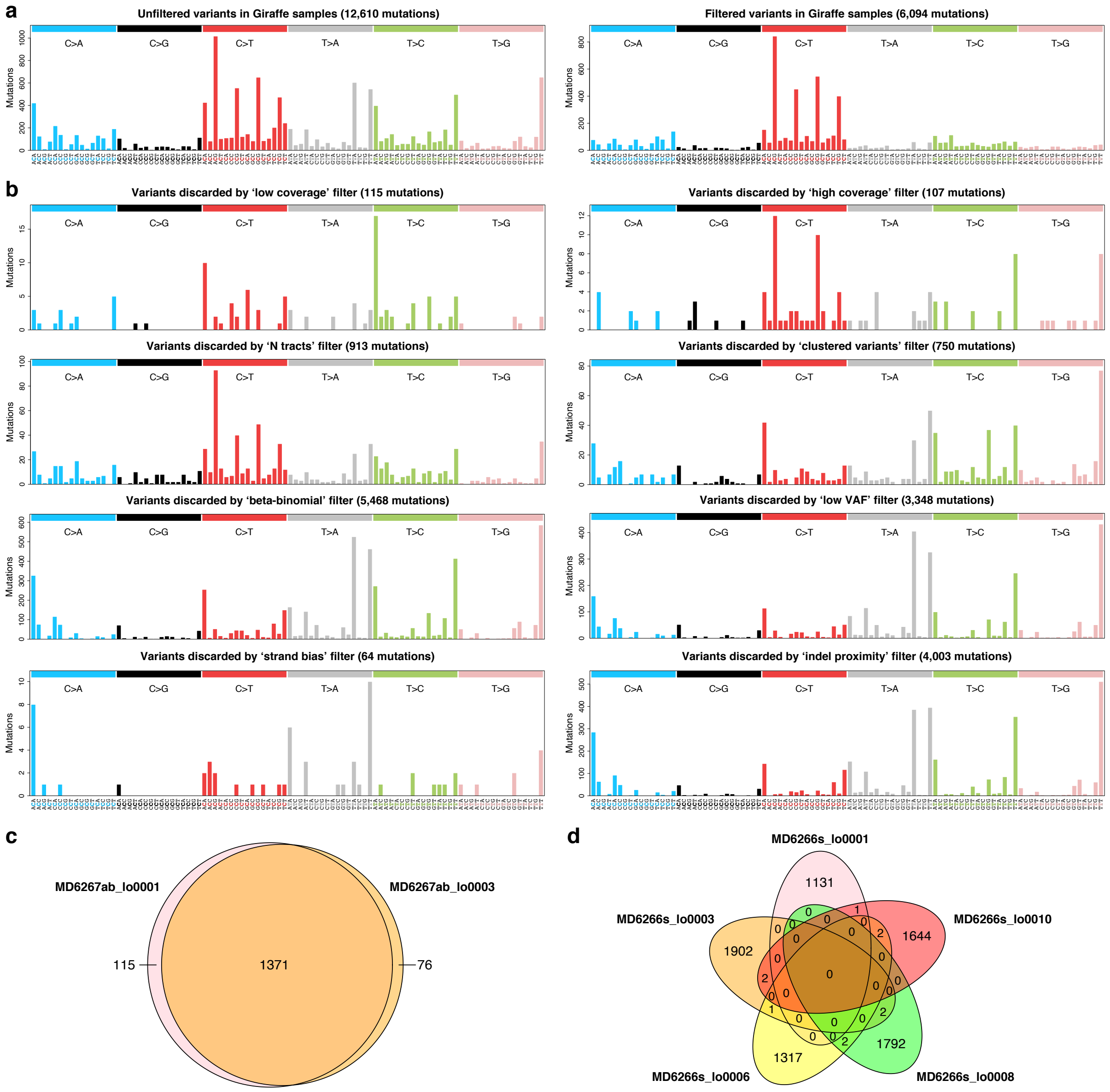

Supplementary Figure 1. Assessment of variant calling and filtering. a, Spectra of single-base substitution calls before (left) and after application of the final eight variant filters, across all giraffe samples. Note that the set of 'unfiltered' variants (left) has gone through the three early filters named 'quality flag filter', 'alignment quality filter' and 'hairpin filter' (Methods). **b**, Spectra of substitution calls flagged as artefactual by each of the final eight variant filters, across all giraffe samples. Note that sets of calls flagged by different filters are not mutually exclusive. **c**, Venn diagram showing the number of substitution calls shared between two LCM sections from the same mouse colorectal crypt. **d**, Venn diagram showing the numbers of substitution calls shared between five different colorectal crypts from the same mouse.

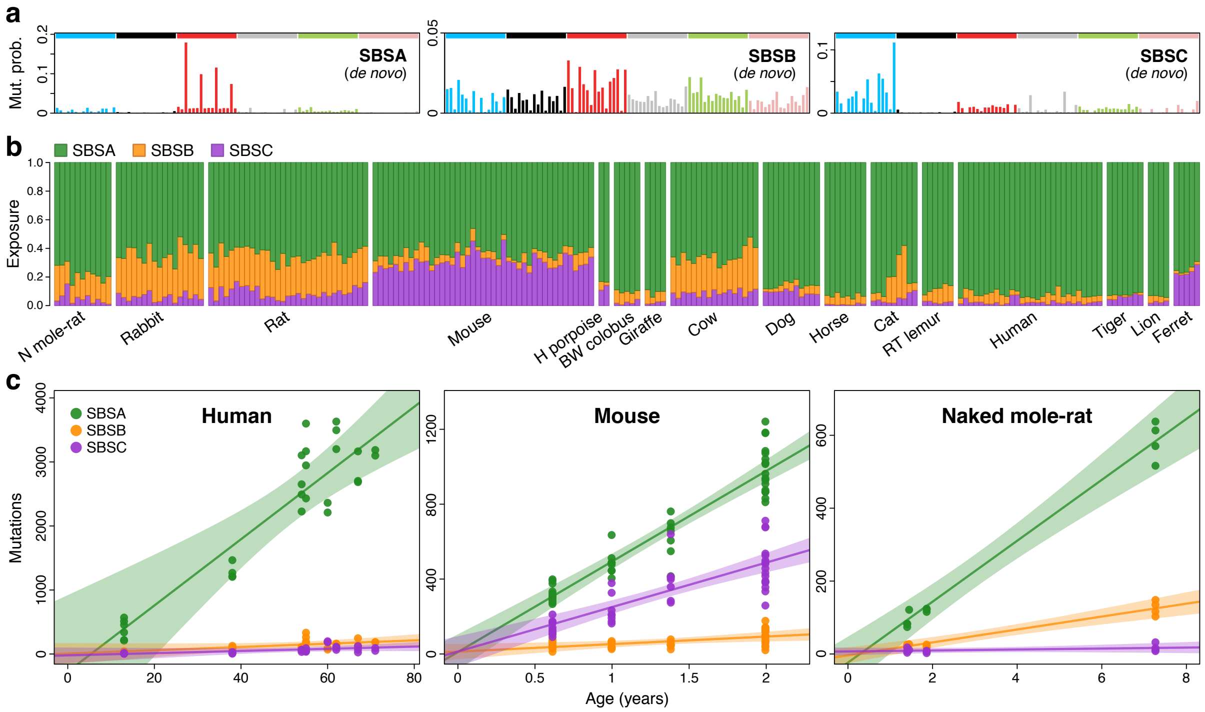

Supplementary Figure 2. Mutational signatures and exposures as inferred *de novo*. a, Mutational signatures inferred de novo from the species mutational spectra shown in **Fig. 2a**. Signatures are shown in a human-genome-relative representation. SBSA is the de novo equivalent of COSMIC signature SBS1 (**Fig. 2b**). **b**, Exposure of each sample to each of the mutational signatures shown in a. Samples are arranged horizontally as in **Fig. 1b**. **c**, Regression of signature-specific mutation burdens on individual age for human, mouse and naked mole-rat samples. Regression was performed using mean mutation burden per individual. Shaded areas indicate 95% confidence intervals of the regression lines. BW, black-and-white; H, harbour; N, naked; RT, ring-tailed.

Supplementary Table legends

Supplementary Table 1. Species information. For each of the (sub)species in the study, the table provides: common name, scientific name, number of individuals in the study, number of colorectal crypts sequenced, range of individual ages, and source institution.

Supplementary Table 2. Sample information. For each colorectal crypt sample in the study, the table provides: sample ID, individual ID, species name, matched normal sample ID, matched normal sample type, and median sequencing depth.

Supplementary Table 3. Mutation rate and burden regression coefficients per species. For each species in the study (except harbour porpoise), the table provides the mean observed values of the rate of somatic substitutions per genome per year, and point estimates and 95% confidence intervals for simple linear regression of mean substitution burdens per individual on individual ages. The estimated regression slopes correspond to the estimated mutation rate per year for each species. Estimates are provided for constrained-intercept linear models applied to all species, and for free-intercept linear models applied to the eight species with at least three individuals.

Supplementary Table 4. Somatic mutation burdens, rates and signature exposures. For each colorectal crypt sample in the study, the table provides: sample ID, individual ID, species name, individual age, total genome size, coding genome size, analysable genome size, analysable mtDNA size, mutational signature exposures (SBS1, SBSB, SBSC); somatic mutation burdens per genome for single-base substitutions, indels, signature-specific substitutions (SBS1, SBSB, SBSC), and mtDNA mutations; and somatic mutation rates per genome per year for single-base substitutions, indels, signature-specific substitutions (SBS1, SBSB, SBSC), and mtDNA mutations.

Supplementary Table 5. Reference genome information. For each species in the study, the table provides: reference genome version used, reference mtDNA sequence used, Ensembl genome annotation version used (where applicable), reference genome file source, reference mtDNA file source, reference genome file URL, reference mtDNA file URL.

Supplementary Table 6. Life history data. For each species in the study (except harbour porpoise), the table provides: adult mass (g), basal metabolic rate (W), litter/clutch size, maximum longevity (years); and maximum likelihood estimate and 95% confidence limits for the estimated 80% lifespan, together with the corresponding sample size. The source of each estimate is given in brackets.

Supplementary Table 7. Number of cell divisions per lifespan. For mouse, rat and human, the table provides: estimated rate of colorectal cell division (hours), estimated lifespan (years), estimated number of cell division at the end of lifespan, estimated number of mutations per cell division (obtained using our mutation rate estimates), and the reference for the cell division rate. Two estimates of cell division rate are included for human.
