# Supplementary material for: Somatic mutation rates scale with lifespan across mammals
Source: Nature. 2022 Apr 13;604(7906):517–24. doi: 10.1038/s41586-022-04618-z (PMC9021023; doi:10.1038/s41586-022-04618-z)
Supplement: Supplementary file 2 — Reporting Summary [file 41586_2022_4618_MOESM2_ESM.pdf]

Corresponding author(s): Alex Cagan, Iñigo Martincorena

Last updated by author(s): Jan 11, 2022

Reporting Summary

Nature Research wishes to improve the reproducibility of the work that we publish. This form provides structure for consistency and transparency in reporting. For further information on Nature Research policies, see our Editorial Policies and the Editorial Policy Checklist.

Statistics

For all statistical analyses, confirm that the following items are present in the figure legend, table legend, main text, or Methods section.

n/a	Confirmed	
☐	☒	The exact sample size (*n*) for each experimental group/condition, given as a discrete number and unit of measurement
☐	☒	A statement on whether measurements were taken from distinct samples or whether the same sample was measured repeatedly
☐	☒	The statistical test(s) used AND whether they are one- or two-sided<br>*Only common tests should be described solely by name; describe more complex techniques in the Methods section.*
☐	☒	A description of all covariates tested
☐	☒	A description of any assumptions or corrections, such as tests of normality and adjustment for multiple comparisons
☐	☒	A full description of the statistical parameters including central tendency (e.g. means) or other basic estimates (e.g. regression coefficient) AND variation (e.g. standard deviation) or associated estimates of uncertainty (e.g. confidence intervals)
☐	☒	For null hypothesis testing, the test statistic (e.g. *F*, *t*, *r*) with confidence intervals, effect sizes, degrees of freedom and *P* value noted<br>*Give P values as exact values whenever suitable.*
☐	☒	For Bayesian analysis, information on the choice of priors and Markov chain Monte Carlo settings
☐	☒	For hierarchical and complex designs, identification of the appropriate level for tests and full reporting of outcomes
☒	☐	Estimates of effect sizes (e.g. Cohen's *d*, Pearson's *r*), indicating how they were calculated

Our web collection on statistics for biologists contains articles on many of the points above.

Software and code

Policy information about availability of computer code

Data collection	No software was used
Data analysis	Most analyses have been done with bespoke pipelines, deposited in Zenodo (https://doi.org/10.5281/zenodo.5554801) and GitHub (https://github.com/baezortega/CrossSpecies2021). Analyses in R were done with R v3.6.2. R packages used include: caper (v1.0.1), deepSNV (v1.32.0), dNdScv (v0.0.1.0), MutationalPatterns (v1.12.0), nlme (v3.1-137), sigfit (v2.1.0). Our pipeline makes use of the software BWA (v0.7.17-r1188), CaVEMan (v1.13.15), Pindel (v3.3.0), bedtools (v2.17.0), biobambam2 (v2.0.86), Indelwald (v24/09/2021).

For manuscripts utilizing custom algorithms or software that are central to the research but not yet described in published literature, software must be made available to editors and reviewers. We strongly encourage code deposition in a community repository (e.g. GitHub). See the Nature Research guidelines for submitting code & software for further information.

Data

Policy information about availability of data

All manuscripts must include a data availability statement. This statement should provide the following information, where applicable:
- Accession codes, unique identifiers, or web links for publicly available datasets
- A list of figures that have associated raw data
- A description of any restrictions on data availability

DNA sequence data have been deposited in the European Genome-Phenome Archive (EGA; https://ega-archive.org) under overarching accession EGAD00001008032. Preprocessed data files used in the analyses have been deposited in Zenodo (https://doi.org/10.5281/zenodo.5554777). Human DNA sequence data from a previous study (Lee-Six et al., 2019) are deposited in EGA (accession EGAD00001004192).

Field-specific reporting

Please select the one below that is the best fit for your research. If you are not sure, read the appropriate sections before making your selection.

☒ Life sciences ☐ Behavioural & social sciences ☐ Ecological, evolutionary & environmental sciences

For a reference copy of the document with all sections, see nature.com/documents/nr-reporting-summary-flat.pdf

Life sciences study design

All studies must disclose on these points even when the disclosure is negative.

Sample size	Information on sample sizes is provided for all analyses. We selected samples from available individuals attempting to span a wide range of ages. The requested sequencing coverage (40x) was chosen to achieve high sensitivity and specificity for clonal somatic variants.
Data exclusions	We excluded 41 samples due to evidence of polyclonality or poor sequencing quality. The criteria used to assess sample clonality and quality are explained in the Methods, 'Sample filtering' section.
Replication	To confirm the reproducibility of somatic variant calls we used laser capture microdissection to microdissect and sequence two sections from the same mouse colorectal crypt. Both sections were submitted for independent library preparation, genome sequencing, variant calling and filtering using our pipeline. The vast majority of somatic substitution calls were shared between both sections (see Methods & Supplementary Figure 1c), confirming the replicability of our somatic variant calls.<br>Mutation signature extraction was performed with two different methods that gave broadly consistent results (Methods).<br>The main regression results were replicated using a number of different regression models (Methods).
Randomization	Our study design did not involve experimental groups. Covariates were controlled for by including them in our regression models (Methods).
Blinding	We did not apply randomization because we did not have a case/control study design or treatment groups. While sample metadata (such as animal age) did not inform the variant calling process, which was applied in an identical manner for all samples, there was no enforced blinding procedure.

Reporting for specific materials, systems and methods

We require information from authors about some types of materials, experimental systems and methods used in many studies. Here, indicate whether each material, system or method listed is relevant to your study. If you are not sure if a list item applies to your research, read the appropriate section before selecting a response.

Materials & experimental systems

n/a	Involved in the study	
☒	☐	Antibodies
☒	☐	Eukaryotic cell lines
☒	☐	Palaeontology and archaeology
☐	☒	Animals and other organisms
☒	☐	Human research participants
☒	☐	Clinical data
☒	☐	Dual use research of concern

Methods

n/a	Involved in the study	
☒	☐	ChIP-seq
☒	☐	Flow cytometry
☒	☐	MRI-based neuroimaging

Animals and other organisms

Policy information about studies involving animals; ARRIVE guidelines recommended for reporting animal research

Laboratory animals	We did not maintain laboratory animals specifically for this study. Tissue samples of rabbit were purchased from a commercial provider. Samples from mouse, rat and naked mole rat were obtained from collaborators maintaining these lines for other research projects. Samples from other species were collected opportunistically at necropsy. The species, strains, individuals, age and source are reported in extended data tables 1 and 4.
Wild animals	Sample materials were collected from a stranded wild harbour porpoise by the UK Cetacean Strandings Investigation Program (CSIP). The individual was deceased at the time of sample collection. CSIP is funded by Defra and the devolved administration to investigate and document strandings of cetaceans and other marine life around the UK coastline.
Field-collected samples	The study did not involve animals collected from the field.
Ethics oversight	All animal samples were obtained with the approval of the local ethical review committee (AWERB) at the Wellcome Sanger Institute and those at the holding institutions.

Note that full information on the approval of the study protocol must also be provided in the manuscript.
